# Short Leukocyte Telomeres, But Not Telomere Attrition Rates, Predict Memory Decline in the 20-Year Longitudinal Betula Study

**DOI:** 10.1093/gerona/glaa322

**Published:** 2020-12-28

**Authors:** Sara Pudas, Maria Josefsson, Annelie Nordin Adolfsson, Mattias Landfors, Karolina Kauppi, Line Marie Veng-Taasti, Magnus Hultdin, Rolf Adolfsson, Sofie Degerman

**Affiliations:** 1 Department of Integrative Medical Biology, Umeå University, Sweden; 2 Center for Ageing and Demographic Research, Umeå University, Sweden; 3 Department of Clinical Sciences, Umeå University, Sweden; 4 Department of Medical Biosciences, Pathology, Umeå University, Sweden; 5 Department of Medical Epidemiology and Biostatistics, Karolinska Institute, Stockholm, Sweden; 6 Department of Psychology, Umeå University, Sweden; 7 Department of Clinical Microbiology, Umeå University, Sweden

**Keywords:** Cognitive aging, Leukocyte telomere length, Longitudinal, Memory, Population-based

## Abstract

Leukocyte telomere length (LTL) is a proposed biomarker for aging-related disorders, including cognitive decline and dementia. Long-term longitudinal studies measuring intra-individual changes in both LTL and cognitive outcomes are scarce, precluding strong conclusions about a potential aging-related relationship between LTL shortening and cognitive decline. This study investigated associations between baseline levels and longitudinal changes in LTL and memory performance across an up to 20-year follow-up in 880 dementia-free participants from a population-based study (mean baseline age: 56.8 years, range: 40–80; 52% female). Shorter baseline LTL significantly predicted subsequent memory decline (*r* = .34, 95% confidence interval: 0.06, 0.82), controlling for age, sex, and other relevant covariates. No significant associations were however observed between intra-individual changes in LTL and memory, neither concurrently nor with a 5-year time-lag between LTL shortening and memory decline. These results support the notion of short LTL as a predictive factor for aging-related memory decline, but suggest that LTL dynamics in adulthood and older age may be less informative of cognitive outcomes in aging. Furthermore, the results highlight the importance of long-term longitudinal evaluation of outcomes in biomarker research.

Telomeres are dynamic DNA complexes located at the ends of chromosomes, that protect the DNA from degradation. They shorten with every cell division, and therefore also with aging (1). Critically short or sufficiently damaged telomeres induce apoptosis or cellular senescence, but if senescence is bypassed, the unprotected telomeres may cause chromosomal instability due to DNA recombination and end-joining events (1). Experimental evidence shows that telomere shortening is a determinant of tissue-specific and systemic aging (2–4). TL is thought to be determined by a complex interaction of heritable (5), environmental, and lifestyle factors (2). Examples of the latter are life stress (6), lower socioeconomic status (7), obesity, and smoking (8), which have been associated with TL cross-sectionally, although not always consistently across studies (9). Such factors may promote oxidative stress and inflammation, 2 biological mechanisms shown to accelerate telomere shortening (10,11). Collectively, these observations have led to the postulation that TL, often measured in easily accessible tissues such as blood leukocytes, is a biomarker of cellular aging. Indeed, shorter leukocyte telomere length (LTL) has been linked to mortality (12) and age-associated morbidities including cancers and cardiovascular diseases (1), as well as neurocognitive disorders such as cognitive decline (13,14), mild cognitive impairment (15), and Alzheimer’s disease (16).

The biological mechanisms behind a potential link between LTL and neurocognitive function remain elusive so far, but reasonably high correlations between TLs in most human tissues suggest that LTL may be a proxy for system-wide TL, including brain TLs (2,17,18). Critically short TL in some brain cell types have been shown to have detrimental effects (19,20), but a direct link to LTL has not yet been demonstrated. Indirect associations between LTL and neurocognitive function are more plausible. For instance, peripheral telomere dysfunction may have an indirect causal effect on neurocognitive function, mediated by reduced immune system functioning (21), causing increased morbidity and cascading negative effects on the central nervous system (CNS). Short TL in peripheral or CNS cells could drive inflammatory processes by inducing cellular senescence, which is associated with secretion of pro-inflammatory factors (22,23). A purely correlative association could arise via inflammatory or other causes that have separate adverse effects on LTL and the CNS, or be mediated by telomerase activity or expression (24).

Although the mechanistic link between LTL and neurocognitive function is not completely elucidated, LTL may still be valuable as a biomarker for neurocognitive aging. Several studies have indeed demonstrated that short LTL is associated with lower functioning in various cognitive domains (25–27), although thus far mostly based on cross-sectional data. Cross-sectional designs are insufficient to prove an aging-related relationship between LTL and cognition since any observed associations could have arisen in early-life, or be confounded by non-aging-related differences between older and younger cohorts. A few longitudinal studies have demonstrated associations between baseline LTL and subsequent within-person cognitive change, with follow-up times ranging between 4.3 and 7 years (13,14). However, a more recent large-scale study failed to observe baseline LTL-cognitive change association across 4 prospective cohort studies with 3.9–10.5 years of follow-up for cognition (28). Few studies have investigated within-person change in LTL and its potential link to cognition in older age, but one study found an association between LTL attrition across a decade in younger adulthood and global cognition in midlife (48–52 years) (29). More compelling observational evidence for an aging-related association between LTL and healthy cognitive aging would be provided by demonstrating associations between longitudinally estimated changes in LTL and longitudinal cognitive decline in older adults. One study comprising 2 Scottish cohorts failed to demonstrate such an association between changes in LTL and changes in general cognitive function from age 70 to 76 years, or between ages 79 and 92 years (30). Thus, although some evidence links TL and cognitive function in healthy aging, the associations are inconsistent across studies, and mostly based on cross-sectional data.

This study addressed the scarcity of longitudinal research by investigating long-term longitudinal relationships between LTL and memory ability across an up to 20-year follow-up period in 880 nondemented individuals (mean baseline age: 56.8 years). Dementia screening was performed to study the predictive ability of LTL as a biomarker in healthy aging. The statistical model employed (31) enabled simultaneous testing of associations between within-person changes in both LTL and memory, as well as between baseline levels and subsequent changes in the 2 traits. Furthermore, a time-lagged analysis tested the hypothesis that LTL shortening may predict memory decline with a temporal delay. This investigation of joint changes in LTL and memory may provide insights for both the cognitive aging literature and biomarker methodology research.

## Method

### Ethics Statement

The study was conducted following the ethical standards in the Declaration of Helsinki and was approved by the Regional Ethical Review Board in Umeå (from January 1, 2019 incorporated into the Swedish Ethical Review Authority). Informed consent was obtained from all study participants.

### Participants

The study was based on 2 cohorts (samples 1 and 3) of the longitudinal population-based Betula study (32). The cohorts, recruited in 1988–1990 and 1993–1995, respectively, comprised 1963 individuals in total, who were re-invited for follow-up every fifth year. At least one LTL measurement was available for 1751 individuals. The main reasons for missingness were availability of blood samples, which were not collected for some participants, while for others no blood remained in the saved samples at the time of LTL analyses; and drop-out of the study before test wave 2, which was the baseline for LTL measurements (*n* = 164 individuals from sample 1). To promote the stability of our statistical models, only individuals with at least 2 contemporaneous measurements for both LTL and cognition, *n* = 1133, were considered for the current study. Of these, 190 individuals were excluded due to developing dementia at some point of the study, leaving 943 individuals in the analytical sample. The final adjusted model comprised 880 individuals due to missing data for one or more covariate (*n* = 28 missing for *APOE* status; *n* = 20 for lymphocyte proportion; *n* = 8 for education; and *n* = 8 for a health complaints-variable). A flowchart of the inclusion process can be found in the supplementary information (Supplementary Figure 1). Demographics and characteristics of the included participants are given in Table 1. Information about participant race was not collected in the Betula study, but the overall racial composition of these age groups in the catchment area at the time of study recruitment would suggest that the vast majority of the participants are White.

**Table 1.** Sample Characteristics

Final Analytical Sample	
*n*	880
Female	52%
Number of LTL measurements	3.06 (0.91)
Follow-up time for LTL, years	13.34 (5.72)
Age at first LTL measurement, years	56.76 (12.00)
Education, years	11.13 (3.96)
Number of health complaints	3.25 (2.62)
Lymphocyte proportion	0.32 (0.07)
Heart disease or stroke*	60%
Ever smoked*	55%
Obese (BMI ≥ 30)	14%
Self-reported general stress	31%

*Notes*: Numbers represent sample means or percentages, with standard deviations in parentheses. Data were obtained from the baseline LTL time-point, except covariates marked with an asterisk (*), which indicate the self-reported presence of disease/trait at any time-point during study participation. BMI = Body mass index; LTL = Leukocyte telomere length.

### Dementia Assessment

In brief, the dementia diagnoses were based on comprehensive clinical data from repeated evaluations of multidisciplinary medical records, as well as outcomes of the Betula health and memory assessments. The diagnostic method allowed changes in individuals’ symptoms and functional levels to be followed over time and to be weighed into the final diagnosis. The evaluations were coordinated by the coauthor R.A. throughout the study period, applying DSM-IV classification core criteria for dementia. For full assessment procedures, see (33).

### Memory Assessment

Memory was measured through the summation of 5 verbal memory variables: (i) immediate free recall of 16 imperative verb–noun sentences that were enacted by the participant, (ii) delayed cued recall of nouns from the previously enacted sentences, (iii) immediate free recall of 16 verbally and visually presented verb–noun sentences, (iv) delayed cued recall of nouns from the previously presented sentences, and (v) immediate free recall of 12 verbally presented nouns. Exact testing procedures have been described previously (32). Verbal memory was chosen as the cognitive outcome measure because it had been consistently and comprehensively assessed with multiple measures throughout the study period; been shown to be age-sensitive (34); and been validated against other traits in previous work (34,35). For reliability of the included variables, please refer to (34).

### Blood Sampling and DNA Extraction

Sampling of peripheral blood was initiated at the second test wave (T2) and thereafter performed at each test occasion (T3-T6), carried out at random over the day, in a non-fasting condition. The blood samples, comprising whole blood at T2-T3 and buffy coat at T4-T6, were stored in low-temperature freezers (−80°C) until DNA-extraction. DNA was extracted in 2011–2015 with the Kleargene XL blood DNA extraction kit (http://www.lgcgenomics.com), and quantified by UV spectrophotometry, followed by DNA concentration normalization to 30 ng/µL, and storage at −80°C until TL measurement.

### Telomere Length Measurement and Normalization

Relative LTL was determined by the quantitative-PCR method described by Cawthon (36), with minor modifications, as described in ref. (37). All samples were analyzed during a 3-month period in 2014. The interassay coefficient of variation was 4%, and the intra-assay coefficient of variation was 2%, determined by including a reference sample DNA in every run. Each DNA sample was analyzed in triplicate wells in separate telomere (TEL) and single-copy gene HBB (hemoglobin subunit beta, Gene ID:3043) reactions on an ABI 7900HT instrument (Applied Biosystems). T/S (TEL/HBB) values were calculated by the 2^−ΔCt^ method, where ΔCt = Ct_TEL_-Ct_HBB_. The relative LTL value for each sample was generated by dividing each sample’s T/S value with the T/S value of a reference cell line DNA (CCRF-CEM; human acute lymphoblastic leukemia) that was included in all runs.

The obtained LTL values were normalized across plates to adjust for between-run variation and potential differences between whole blood and buffy coat blood sampling. This was done by subtracting the mean-centered plate effects, estimated through a mixed-effects model with age, gender, age × gender interaction and plate as fixed effects, and individuals as a random effect. After adjustment for plate effects, an ordinary least squares model with age, gender, and age × gender interaction was fitted. Residuals greater than 3.67 times residual standard deviation were flagged as outliers and removed from the data (14 LTL measures).

### Covariates

In addition to the covariates sex, age, and age squared at baseline, several variables previously found to be associative with TL or memory function in aging were evaluated as covariates in an initial model. This was done to establish the independence of possible LTL-memory associations from confounding factors. Most covariates were recorded from the time point of the first TL measurement of each participant. This was true for self-reported years of education, *APOE* ε4- carrier status, stress (a binary variable based on one question about self-reported general stress), number of health complaints (summation of self-reported presence of the following conditions: uneasiness in heart/chest, back pain, stomach pain, constipation, joint pains, skin conditions [eg, itching and varicose ulcers], bronchial discomfort, aching shoulders, arms, or legs, shortness of breath, leg swelling, dizziness, loss of appetite, difficulties urinating, insomnia, fatigue, feelings of depression, feelings of loneliness, anxiety or worry, vision problems, and hearing difficulties), and obesity (coded as a binary variable indicating whether the participant had a measured BMI of 30 or greater). Smoking was coded as a binary variable, indicating whether the participant had reported being a smoker at any time point before or during their participation in the study. Heart disease or stroke was also coded as binary, and reflected whether participants had self-reported the presence of these conditions at any point during study participation. For the memory variable, a dummy variable indicating whether it was the participant’s first test occasion was included to capture practice effects from repeated cognitive testing.

Furthermore, the lymphocyte proportion in the blood sample from the second time-point of the Betula study was included as a covariate intended to capture individual differences in blood cell distributions, which may affect LTL measurements since lymphocytes have shorter TL than granulocytes (30,38). Blood cell proportions were only available from the second time point (T2) of the study, which coincided with the baseline LTL measurement for 69.5% of the sample. For 29.9% of the sample baseline, LTL was measured 5 years after the blood sample used to determine blood cell counts was taken, and for 0.6%, baseline LTL measurement occurred 10–15 years later. However, high within-person stability of lymphocyte proportions across 5 years (partial correlation *r* = .485, *p* < .001, controlling for age, age-squared, and sex) was observed in a subsample of 836 participants from Betula sample 1 who had blood cell proportion data at time points 1 and 2 (T1-T2) of the study. Based on this observation, we decided to include the T2 blood cell proportions as a covariate to at least partially account for individual differences in blood cell proportions. In addition, to capture possible effects of acute or chronic illness on blood cell proportion and/or LTL, the erythrocyte sedimentation rate (available from all time-points) was entered as a time-varying covariate (log-transformed due to skewness) to the statistical models.

### APOE Genotyping

Genotyping for APOE was performed by polymerase chain reaction, as described in Nilsson et al. (39).

### Statistical Modeling

A bivariate linear mixed model (BLMM) framework was applied (for details, see (40)) to test for an association between individual change in memory scores and LTL jointly. The BLMM is an extension of the standard univariate linear mixed model to a bivariate model for 2 outcomes. The bivariate model assumes a linear mixed model for each outcome, and these univariate models are combined through the specification of a joint multivariate normal distribution for all random effects. The correlations between the random effects are obtained from the variance–covariance matrix and summarize how the evolution of memory is associated with the evolution of LTL.

Fixed effects, representing the average among the study population, initially included all the covariates described above as main effects and interacting with time (a linear effect), for each outcome. Random effects comprised an intercept and slope for each outcome and individual, and were assumed to be correlated. The continuous covariates were standardized, to a mean of zero and a standard deviation of one, to avoid multicollinearity and to facilitate interpretation of covariate effects.

The BLMMs were fitted in R (41) using the nlme package. The 95% confidence intervals (CI) of the model parameters and random effects correlations were estimated using 2500 nonparametric bootstrap samples. Inference of the parameters in the models are based on Restricted Maximum Likelihood estimation and allows for an unequal number of measurements per subject and outcome, and is valid if, conditional on the observed data, the nonresponse mechanism is assumed to be missing at random (31).

Backward elimination was used for variable selection from the set of candidate covariates. The algorithm started with a model including all covariates and their interaction with time, and iteratively excluded variables with the largest *p*-value until all variables in the model had a *p*-value < .05. Main covariate effects were kept in the final model if their interaction with time was significant. The Satterthwaite approximation was used to calculate *p*-values in the backward elimination algorithm to reduce computation time.

### Data Availability

Upon request from qualified investigators, pseudonymized data used in this study may be shared for replication purposes, as long as the data transfer is in agreement with European Union legislation on the General Data Protection Regulation and Umeå University data protection policies.

## Results

### Sample Characteristics and Descriptive Data

**Figure 1.**
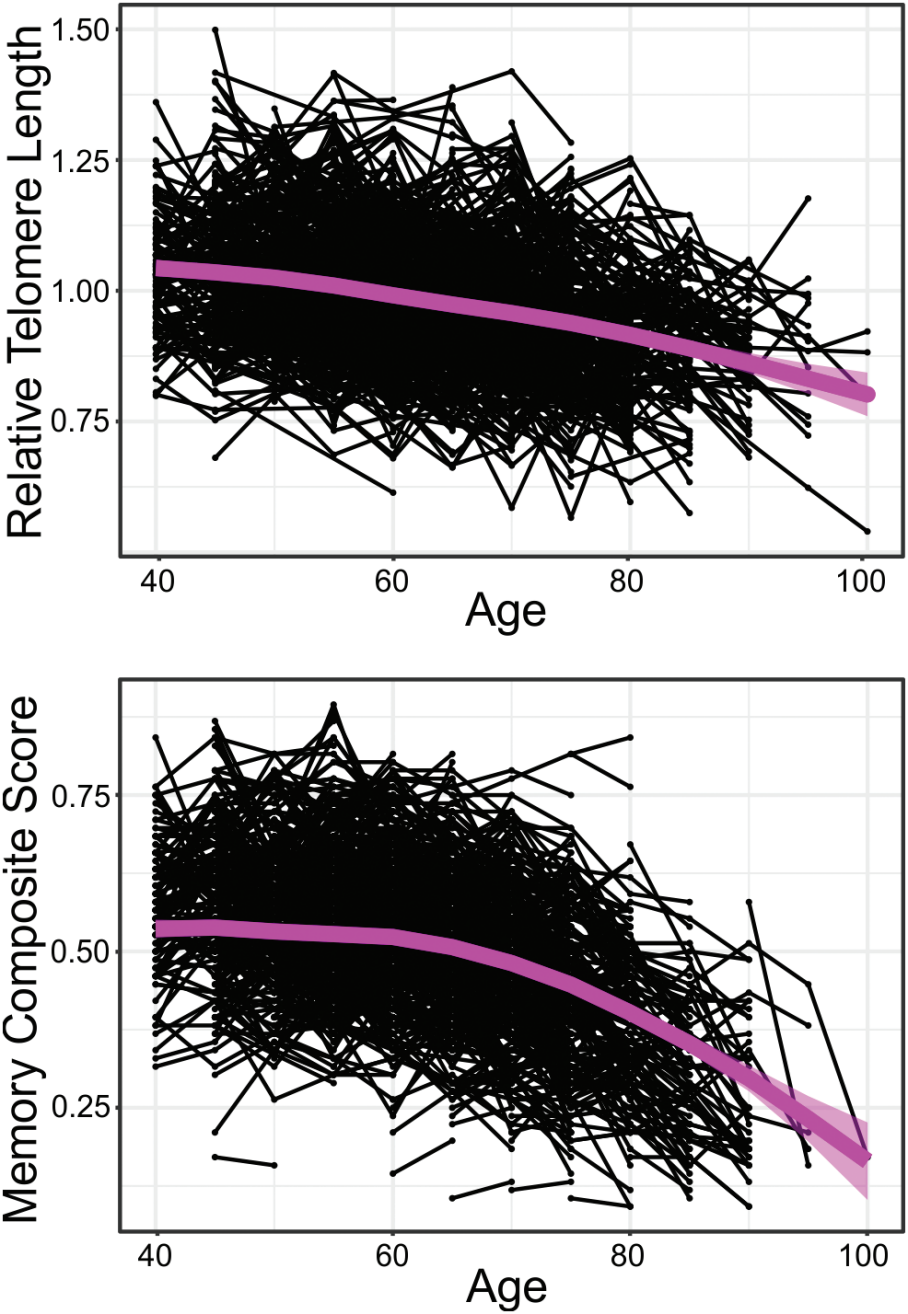
Descriptive data for memory performance and leukocyte telomere length. Memory score is expressed in percent. The unit for telomere length is relative. Black dots indicate individual observations, while black lines denote intra-individual changes over time. The thicker line shows the sample average change based on all observed data, estimated by a generalized additive mixed model.

The main analytical sample consisted of 880 individuals aged 40–85 years (average age: 56.76 years) at baseline LTL measurement (52% female), who had remained dementia-free throughout the study period and contributed at least 2 contemporaneous measurements of LTL and memory. The included individuals contributed on average 3 observations of LTL and memory, measured every fifth year. The average follow-up time was 13 years (*SD* = 5.72, range = 5–20; for a breakdown of LTL follow-up times, see Supplementary Table S1). Other sample characteristics are given in Table 1. Figure 1 shows the raw data for LTL and memory scores, as well as their sample-averaged change trajectories estimated by a generalized additive mixed model (42).

### Model-Estimated Longitudinal Changes and Covariate Effects

Bivariate linear mixed models (BLMMs) were set up with study time as the time scale, and random effects for the intercepts and time effects. Several potentially confounding covariates were evaluated in an initial BLMM (see Methods), including covariate × time interactions, which tested whether the covariate was associated with changes in LTL or memory over time. Backwards elimination was thereafter used to build the final model by iteratively removing covariates and refitting the model until all remaining variables had a *p* < .05. The full results including all covariates are given in Supplementary Table S2.

In the final covariate-adjusted model (Table 2), the average LTL reduction (“Time”-effect) was estimated to −0.018 units per study wave, that is, 5-year time interval, which corresponds to an LTL shortening of 0.35% per year (in relation to the model-estimated intercept). A 1 year increase in baseline age was associated with 0.25% shorter LTL. Also, males had shorter baseline LTL (−2.82%), while higher education was associated with longer baseline LTL (0.97% for 1 *SD* higher education). Self-reported presence of heart disease or stroke was associated with shorter baseline LTL, as was a higher baseline lymphocyte proportion. Finally, as indicated by a significant baseline age by time interaction, individuals who were older at study baseline experienced a more rapid decline in LTL (Table 2). No other covariates were significantly associated with the rate of LTL shortening.

**Table 2.** Covariate Effects on Baseline Levels and Changes in Telomere Length and Memory

Fixed Effects	Estimate (95% CI)
**Telomere length**	
Intercept	1.030 (1.019, 1.041)
Time	−0.018 (−0.020, −0.016)
Baseline age	−0.029 (−0.034, −0.023)
Male	−0.029 (−0.040, −0.019)
Education	0.010 (0.004, 0.016)
Heart disease or stroke	−0.019 (−0.031, −0.008)
Lymphocyte proportion	−0.013 (−0.018, −0.007)
Time × Baseline age	−0.004 (−0.006, −0.002)
**Memory**	
Intercept	0.542 (0.534, 0.550)
Time	−0.017 (−0.019, −0.015)
Baseline age	0.140 (0.095, 0.181)
Baseline age-squared	−0.179 (−0.220, −0.136)
Male	−0.041 (−0.051, −0.032)
Education	0.044 (0.038, 0.050)
*APOE* ε4-carrier	0.005 (−0.006, 0.016)
Lymphocyte proportion	−0.006 (−0.010, −0.001)
Health complaints	−0.001 (−0.006, 0.004)
First test occasion	−0.024 (−0.031, −0.018)
Time × Baseline age	−0.013 (−0.015, −0.011)
Time × *APOE* ε4-carrier	−0.006 (−0.009, −0.002)
Time × Lymphocyte proportion	0.003 (0.001, 0.004)
Time × Health complaints	−0.002 (−0.004, −0.001)

*Notes*: Time denotes within-sample longitudinal change, per study wave (ie, 5-year interval). Interactions with time represent significant effects of the respective covariate on telomere length or memory change over time. Continuous covariates are scaled to a mean of zero and a standard deviation of one. CI = Confidence interval.

For memory, the fully-adjusted model indicated a significant reduction of memory by 1.7% per 5-year period, or −0.34% per year (“Time”-effect, Table 2). Baseline memory level was significantly associated with the covariates baseline age, age-squared, sex, education, and the dummy variable for first memory test occasion, all with effects in the expected direction (Table 2). The rate of memory decline was significantly associated with higher baseline age, presence of the *APOE* ε4-allele, and more health complaints at baseline, as indicated by significant interactions between these variables and the time effect in the model. In addition, higher lymphocyte proportion had a negative association with baseline memory performance and a positive association with the rate of memory decline. This indicates that individuals with a higher proportion of lymphocytes at baseline had a lower than average memory performance at study baseline, but also showed less memory decline over time.

### Concurrent Associations Between LTL and Memory Levels and Changes

Associations between intercepts and changes in LTL and memory were estimated via correlations between the random effects for the intercept and time effects in the BLMM, and are given in Table 3 (histograms of distributions of random effects are provided in Supplementary Figure 2). Notably, shorter baseline LTL was associated with accelerated memory decline (*r* = .34, 95% CI: 0.06, 0.82). The association between change in LTL and change in memory, across the 20-year study period, was however not significant (*r* = .25, 95% CI: −0.68, 0.91). A positive association between baseline LTL and LTL slope (*r* = .56, 95% CI: 0.03, 0.97) was also observed, indicating that individuals with shorter initial LTL experience more LTL shortening. No other associations were significant.

**Table 3.** Associations Between Baseline Levels and Changes in Telomere Length and Memory

	Concurrent	Time-lagged
	Correlation (95% CI)	Correlation (95% CI)
Baseline LTL – Baseline MEM	0.043 (−0.031, 0.110)	0.081 (0.009, 0.154)
Baseline LTL – LTL Change	0.559 (0.027, 0.966)	−0.100 (−0.215, 0.046)
Baseline LTL – MEM Change	0.340 (0.060, 0.818)	0.111 (−0.097, 0.515)
Baseline MEM - LTL Change	−0.239 (−0.691, 0.182)	−0.057 (−0.175, 0.059)
Baseline MEM – MEM Change	0.283 (−0.036, 0.841)	−0.025 (−0.238, 0.418)
LTL Change – MEM Change	0.247 (−0.680, 0.910)	−0.106 (−0.521, 0.256)

*Note*: CI = Confidence interval; LTL = Leukocyte telomere length; MEM = Memory.

As a sensitivity analysis, we repeated the main analysis including also the dementia cases with complete covariates (*N* = 170 cases; *N* = 1050 in total). The association between baseline LTL and memory change did not change, *r* = .34 (95% CI: 0.14, 0.62), and the association between change in LTL and change in memory remained nonsignificant (*r* = −0.12, 95% CI: −0.69, 0.68). Although this analysis was somewhat limited by the expected higher drop-out rates among individuals with dementia, the result suggests that the nonsignificant change–change association was not a side effect of excluding lower-performing individuals with preclinical or clinical neurodegenerative disorders.

### Time-Lagged Associations Between LTL and Memory Levels and Changes

To test the hypothesis that LTL change may predict memory change with a delay, we performed a 5-year time-lagged analysis on the main analytical sample (*n* = 880). The 5-year delay was motivated by the 5-year interval between test occasions in our dataset. Thus, we tested whether LTL change between study waves 1–4 predicted memory change between waves 2–5. As given in Table 3, the change-change association remained nonsignificant (*r* = −.11, 95% CI: −0.52, 0.26). This model indicated a small, but significant association between baseline LTL and memory measured 5 years later (*r* = .08, 95% CI: 0.01, 0.15), which is in line with baseline LTL predicting memory change in the original model of concurrent associations. However, in the time-lagged model, baseline LTL was no longer significantly associated with the subsequent rate of memory change (*r* = .11, 95% CI: −0.11, 0.48). To test whether this effect reduction might be driven by the shorter follow-up for memory in the time-lagged model (15 years) compared with the contemporaneous model (20 years), we performed a sensitivity analysis removing the last time point in the original model to equate follow-up times. The analysis showed that baseline LTL no longer significantly predicted subsequent memory change when follow-up time was restricted to 15 years (*r* = .16, 95% CI: −0.13, 0.72). This demonstrates the importance of sufficiently long follow-up times to detect associations between LTL and cognitive aging outcomes.

## Discussion

This study found a significant positive association between baseline LTL and subsequent 20-year memory change among 880 cognitively healthy adults and older individuals from a population-based study. Thus, shorter LTL at study baseline was associated with more memory decline. No association could, however, be demonstrated between intra-individual changes in LTL and concurrent or time-lagged changes in memory function across the 20-year study period. The observed association between baseline LTL and memory change was independent of a number of covariates previously associated with LTL and neurocognitive health in aging. Collectively, these results suggest that short TL, established earlier in life, might be a predictive factor for poor neurocognitive outcomes in aging, but that subsequent telomere attrition in adulthood and old age does not track linearly with concomitant or 5-year time-lagged memory decline. This pattern of findings aligns with recent observations of null associations between adulthood LTL shortening and other LTL-related traits (43,44).

Our main finding, a significant association between baseline LTL and subsequent memory decline, is in line with some prior large-scale longitudinal studies observing associations with baseline LTL and subsequent changes on a screening test for dementia (13), and general cognition and verbal memory (14). Other longitudinal studies have, however, failed to observe such effects (28,45), although one of these had a 4-year follow-up time only, and the other observed a nonsignificant trend (*p* = .073) for a positive meta-analytic association between baseline LTL and cognitive decline across 4 studies. Factors such as sample size and characteristics, type of cognitive measure and its reliability, as well as follow-up length, could account for divergent findings. Given the relatively small effect size of LTL- cognition associations observed here and previously (28), large sample sizes and sufficiently long follow-ups are likely key factors for observing associations. This study’s 20-year follow-up time likely contributed to our ability to detect the association, which is supported by the sensitivity analysis showing a reduction of the baseline LTL—memory change association when restricting follow-up to 15 years. Thus, our findings demonstrate the relevance of LTL in relation to long-term cognitive outcomes, even in the absence of neurocognitive disorders. Although only memory performance was considered here, due to being the most reliable cognitive measure available, meta-analytic evidence showing 60%–70% of the variance in change is shared across different cognitive domains (46) makes it difficult to conclude that memory decline is more strongly related to LTL than to decline in other cognitive abilities.

In line with the literature (1,18), we observed significant age-related TL shortening, both cross-sectionally, as a significant effect of age on baseline LTL, and longitudinally, as a significant within-person decline. The longitudinal rate of LTL decline was numerically larger than the cross-sectional age effect (−0.35 vs −0.25%). Despite this, the rate of LTL decline was not significantly associated with the rate of memory decline. This finding concurs with the only other longitudinal study, to our knowledge, that has investigated change-change associations between LTL and cognition in aging (30). Here, we also found no associations between LTL shortening and 5-year time-lagged memory decline. While these null findings cannot prove that an association does not exist, the pattern of findings aligns with recent studies reporting null associations between adulthood LTL shortening and other LTL-related traits such as smoking (44) or carotid stenosis (43), that nevertheless showed associations with baseline LTL. These findings parallel the observation in birds that early-life TL predicted life span more robustly than later-life TL, or adulthood TL shortening (47). Assuming that LTL may have an active role in age-related disorders, others have also argued that the influence of LTL dynamics in adulthood or old age on age-related disease outcomes may be small compared to LTL at birth and its shortening in childhood and youth (48). However, it should be considered that the magnitude of LTL shortening in adulthood may be small compared to individual differences established in youth, as indicated by a longitudinal study of 4 cohorts (*n* = 1156; mean ages: 30–75 years), in which very few individuals changed their rank order for LTL across 12 years (49). Small absolute LTL changes, in combination with measurement error inherent in LTL measurements (50), may also impede detection of significant associations with LTL change, both here and in other studies. Nevertheless, collectively, these observations suggest that baseline LTL may be a more useful biomarker than adulthood LTL dynamics, although our current data can only speak to memory decline.

Our covariate associations also suggest scarcity of LTL-change relations, since only age and baseline LTL were significantly associated with LTL change. Although we did not have hypotheses about specific covariate effects, we note that our baseline LTL covariate associations replicate several common observations from the literature. LTL was shorter in men, (12,13,38), and those with cardiovascular diseases (1), and longer in individuals with higher education, possibly reflecting higher socioeconomic status (7). Higher lymphocyte proportion was associated with shorter baseline LTL, which reflects differential TL in leukocyte subtypes due to differences in replicative histories (38). The positive association between baseline LTL and subsequent LTL change seen here contrasts with some previous studies that have observed negative associations between LTL at baseline and LTL changes. Negative associations could likely be ascribed to statistical regression-to-the mean effects driven by measurement error (51), which our use of a linear mixed modeling approach may have counteracted (52). A positive association between baseline and change could also reflect the influence of unmeasured factors such as inflammation, which are likely to affect both baseline levels and changes in LTL. For memory, several covariates were associated with both baseline levels and longitudinal changes, all with effects that would be expected based on the literature. One exception was the lymphocyte proportion, which showed an unexpected negative association with baseline memory, as well as a positive association with memory change. Since the lymphocyte proportion decreases with age and changes in the presence of acute and chronic illnesses, it is plausible that associations with memory function could arise. However, the mechanism or mechanisms behind the associations with memory here are difficult to explain. Importantly, however, by including lymphocyte proportion as a covariate in our statistical model, we minimized the risk of it acting as a confounder of the association between LTL and memory.

Given the observation of relative stability of individual differences in LTL in healthy adults (49), and the scarcity of evidence for factors associated with the rate of LTL change in adulthood (43,44), it is worthwhile briefly considering potential origins of individual differences in baseline LTL that predicted memory decline in the current study. Since the estimated heritability for LTL is high (>0.6, (5)), they are likely partially heritable, and considerable between-person differences in LTL exist already at birth (53). Furthermore, that adulthood LTL differences have an early-life origin is also consistent with that most LTL shortening occurs in childhood, due to the growing body and increase in blood volume (54). Lifestyle and environmental factors such as obesity (55) and air pollution exposure (56) have also been associated with shorter LTL already in childhood. In fact, studies comparing the timing of exposures to factors such as air pollution (56) or stress (57), have reported larger associations between childhood or early-life exposures and adulthood LTL, than concurrent adulthood exposures. Collectively these studies, and the relative scarcity of factors associated with LTL rate of change in adulthood here and in previous studies, suggest that early-life individual differences in LTL could underlie some of the differences in baseline LTL observed in our sample, and thus be a predictive marker of the poorer age-related cognitive outcomes we observed.

The merits of the current study include the unusually long follow-up time, a well-characterized and reasonable large population-based sample, as well as careful screening for dementia, which minimized the risk of the LTL-cognition association being driven by preclinical neurodegenerative disorders previously associated with shorter LTL (16). However, some limitations should be noted. First, common to all long-term longitudinal studies, selective drop-out of those with poorer health and cognitive outcomes likely lead to an underestimation of LTL and memory changes in our sample, which could have also affected longitudinal LTL-memory associations. This does not however invalidate the significant effects that we did observe, although their effect sizes may have been underestimated. Further, although the sample was well characterized, several covariates were self-reported and could suffer from some degree of error. Another limitation was that we could not control for differences in leukocyte subtype distributions across time, since this information was only available for the second wave of our study. Changes in leukocyte subset proportions (eg, due to acute infections) over time could introduce noise into the estimation of LTL and aggravate detection of meaningful variance in LTL change. However, even in a study where leukocyte subset proportions were accounted for, LTL decline was still not significantly associated with cognitive decline (30). Another limitation is the general measurement imprecision has been reported for qPCR estimation of LTL (50), which also could introduce noise and bias findings toward the null. Nevertheless, qPCR estimation of TL has been shown to have a similar coefficient of variation as the gold standard Southern blot technique (58). Measurement uncertainty associated with these limitations may have constrained our effect sizes, with the consequence that our findings would not hold for statistical correction for multiple comparisons. Replication in larger-scale cohorts is therefore desired, although long-term follow-ups of such datasets are currently lacking.

## Conclusions

The results from this population-based study with a 20-year follow-up time suggest that interindividual variability in baseline LTL may be a more robust predictor of age-related cognitive decline than telomere shortening in adulthood or old age. The fact that individual differences in LTL measured before older adulthood may be predictive for neurocognitive aging even in the absence of neurodegenerative disease is encouraging for the perspective of using LTL as a predictive biomarker, but the relatively small effect sizes and high estimation uncertainty here, and in other studies (28), speak against its use as a standalone biomarker at the present moment. Nevertheless, our finding of a positive association between baseline LTL and subsequent long-term memory decline should encourage continued study of the relationship between LTL and neurocognitive aging. Methodological development of LTL measurement techniques, that allow monitoring TL on single chromosome ends, or measure TL in specific cell subsets, may also increase its feasibility as a biomarker. More studies will also be needed for a better mechanistic understanding of the association between LTL and cognitive and brain aging.

## Funding

This work was supported by a grant from the Swedish Research Council (2018-01729) to S.P. Financial support was also provided through a regional agreement between Umeå University and Västerbotten County Council, grants: RV-735451 (2018–2020); RV-453141 (2015–2017); RV-225461 (2012–2014) and year-wise RV-741571, RV-678571, RV-582111, RV-491371, RV-400741, RV-322831, RV-243741(2012–2018) to R.A.; as well as year-wise RV-932787, RV-865381 and RV-745571 to M.H. K.K. was supported by the Swedish Research Council (2017-03011). This work was also supported by the Medical Faculty at Umeå University, the Kempe Foundation, and Uppsala-Umeå Comprehensive Cancer Consortium.

## Supplementary Material

glaa322_suppl_Supplementary_Materials
