# Supplementary material for: Short Leukocyte Telomeres, But Not Telomere Attrition Rates, Predict Memory Decline in the 20-Year Longitudinal Betula Study
Source: J Gerontol A Biol Sci Med Sci. 2020 Dec 28;76(6):955–63. doi: 10.1093/gerona/glaa322 (PMC8140048; doi:10.1093/gerona/glaa322)
Supplement: glaa322_suppl_Supplementary_Materials [file glaa322_suppl_supplementary_materials.pdf]

Supplementary material for "Short leukocyte telomeres, but not telomere attrition rates, predict memory decline in the 20-year longitudinal Betula study"

Sara Pudas, Maria Josefsson, Annelie Nordin Adolfsson, Mattias Landfors, Karolina Kauppi, Line Marie Veng-Taasti, Magnus Hultdin, Rolf Adolfsson and Sofie Degerman

This document contains eFigure 1, eTable 1, eTable2 and eFigure 2.

eFigure 1 – Flowchart of inclusion/exclusion criteria and associated number of participants

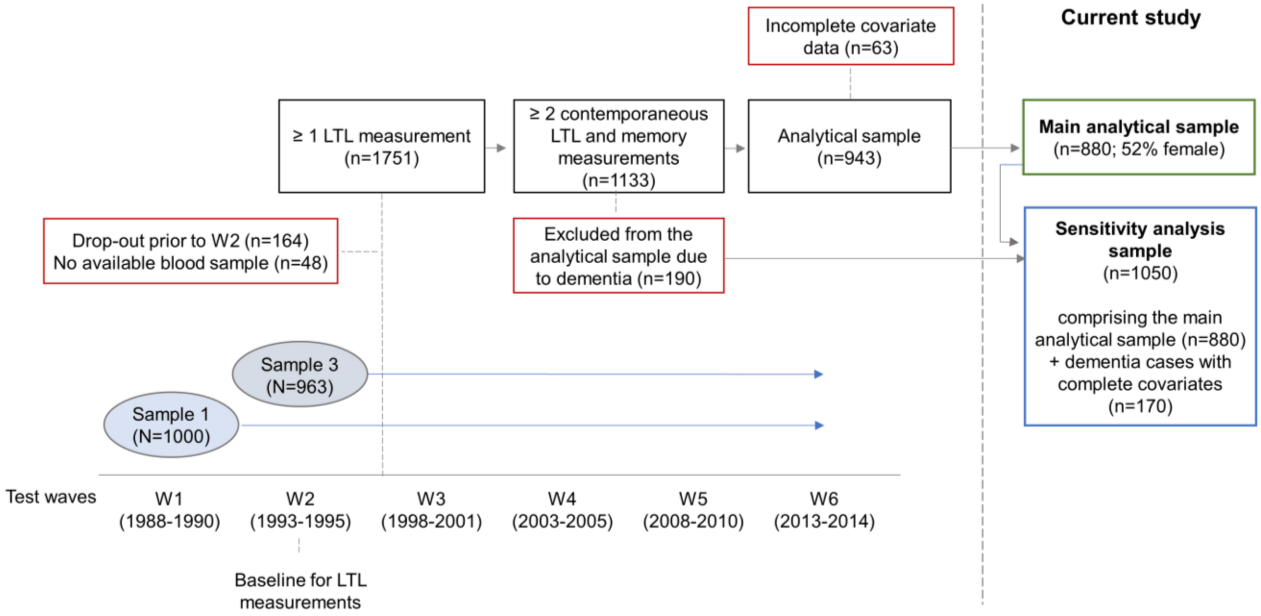

eFigure 1. Flowchart with inclusion and exclusion criteria and associated number of participants for the main analytical sample and the sensitivity analysis sample respectively

eTable 1 – Breakdown of telomere length follow-up times for the included participants

Number of observations, per study follow-up wave				
**Baseline**	**5 years**	**10 years**	**15 years**	**20 years**
880	679	338	536	259
**Number of study participants with different follow-up lengths**				
**5 years**	**10 years**	**15 years**	**20 years**	
219	114	288	259	
**Number of study participants with different numbers of follow-up time-points**				
**2 time-points**	**3 time-points**	**4 time-points**	**5 time-points**	
295	286	251	48	

Note. An inclusion criterion was to have at least 2 measurement points, i.e., a 5-year follow-up length. Due to intermittent missing observations the number of observations at the 15-year follow-up wave was larger than at the 10-year follow-up. Intermittent missing observations also explain the discordance between follow-up lengths and time-points.

eTable 2 – Full bivariate linear mixed model results including all covariates

Fixed effects	Estimate	CI 95%
**Telomere length effects**		
Intercept	1.039	(1.023, 1.055)
Time	-0.018	(-0.023, -0.013)*
Baseline age	-3.8e-05	(-0.047, 0.048)
Baseline age, squared	-0.028	(-0.073, 0.016)
Male	-0.032	(-0.045, -0.020)*
Education	0.007	(0.001, 0.014)*
APOE	-4.0e-04	(-0.015, 0.013)
Lymphocyte proportion	-0.012	(-0.018, -0.006)*
Smoking	-0.007	(-0.019, 0.005)
Obesity at baseline	-0.004	(-0.021, 0.012)
Sedimentation rate	-0.002	(-0.005, 0.002)
Heart disease or stroke	-0.019	(-0.031, -0.007)*
Stress at baseline	-0.005	(-0.019, 0.008)
Health complaints, baseline	-0.004	(-0.010, 0.002)
Time x Baseline age	0.007	(-0.012, 0.026)
Time x Baseline age, squared	-0.012	(-0.032, 0.009)
Time x Male	-4.7e-04	(-0.004, 0.003)
Time x Education	0.001	(-0.001, 0.004)
Time x APOE	-0.001	(-0.005, 0.003)
Time x Lymphocyte proportion	-0.001	(-0.003, 0.001)
Time x Smoking	-0.001	(-0.005, 0.003)
Time x Obesity	-0.001	(-0.008, 0.006)
Time x Heart disease or stroke	0.001	(-0.003, 0.005)
Time x Stress at baseline	0.001	(-0.003, 0.005)
Time x Health complaints at baseline	0.001	(-0.001, 0.003)
**Memory effects**		
Intercept	0.535	(0.521, 0.549)
Time	-0.014	(-0.018, -0.010)*
Baseline age	0.115	(0.069, 0.163)*
Baseline age, squared	-0.155	(-0.202, -0.109)*
Male	-0.041	(-0.051, -0.030)*
Education	0.046	(0.040, 0.051)*
APOE	0.005	(-0.006, 0.017)
Lymphocyte proportion	-0.006	(-0.011, -0.001)*
Smoking	0.002	(-0.008, 0.013)
Obesity at baseline	0.01	(-0.004, 0.024)
Sedimentation rate	-0.003	(-0.005, 9.2e-05)

Memory effects, cont.	Estimate	CI 95%
Heart disease or stroke	0.003	(-0.007, 0.014)
Stress at baseline	0.003	(-0.008, 0.014)
Health complaints at baseline	-0.001	(-0.006, 0.004)
First memory test	-0.023	(-0.029, -0.016)*
Time x Baseline age	0.004	(-0.014, 0.022)
Time x Baseline age, squared	-0.018	(-0.036, 0.002)
Time x Male	-0.002	(-0.005, 0.001)
Time x Education	-0.001	(-0.003, 4.5e-04)
Time x APOE	-0.006	(-0.009, -0.002)*
Time x Lymphocyte proportion	0.002	(0.001, 0.004)*
Time x Smoking	-0.002	(-0.005, 0.001)
Time x Obesity	-0.001	(-0.006, 0.004)
Time x Heart disease or stroke	1.8e-04	(-0.003, 0.003)
Time x Stress at baseline	-0.001	(-0.004, 0.002)
Time x Health complaints at baseline	-0.003	(-0.004, -0.001)*

Note. CI = Confidence interval
* indicates significant fixed effects, estimated using 2,500 non-parametric bootstrap samples.
All continuous covariates were scaled to a mean of zero and a standard deviation of one. For full covariate descriptions, please refer to the methods section in the main text.

eFigure 2 – Histograms of random effects for telomere and memory intercepts and slopes

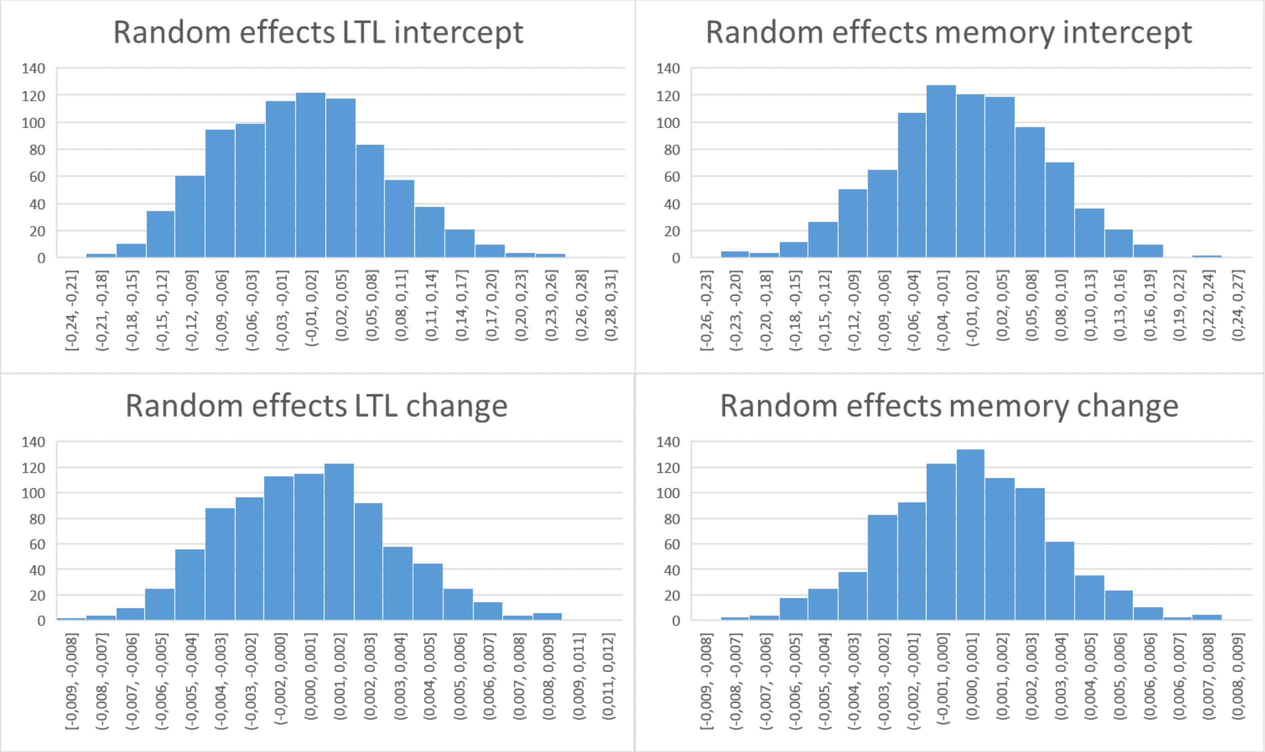

eFigure 2. Histograms of model-estimated random effects for leukocyte telomere length (LTL) and memory intercepts (i.e., baseline levels) and changes over time. Estimates originate from the final covariate-adjusted model reported in Table 2 in the main text, and should be interpreted as individual-level deviations from the fixed-effects intercepts and time-effects (i.e., changes over time) of the models.
